# High-Precision Image Aided Inertial Navigation with Known Features: Observability Analysis and Performance Evaluation

**DOI:** 10.3390/s141019371

**Published:** 2014-10-17

**Authors:** Weiping Jiang, Li Wang, Xiaoji Niu, Quan Zhang, Hui Zhang, Min Tang, Xiangyun Hu

**Affiliations:** 1 GNSS Research Center, Wuhan University, Wuhan 430079, China; E-Mails: wpjiang@whu.edu.cn (W.J.); li.wang@whu.edu.cn (L.W.); zhangquan@whu.edu.cn (Q.Z.); zhanghuiagain@gmail.com (H.Z.); 2 School of Remote Sensing and Information Engineering, Wuhan University, Wuhan 430079, China; E-Mails: min.tang@whu.edu.cn (M.T.); huxy@whu.edu.cn (X.H.)

**Keywords:** image-aided inertial navigation, tightly coupled, observability analysis, high precision

## Abstract

A high-precision image-aided inertial navigation system (INS) is proposed as an alternative to the carrier-phase-based differential Global Navigation Satellite Systems (CDGNSSs) when satellite-based navigation systems are unavailable. In this paper, the image/INS integrated algorithm is modeled by a tightly-coupled iterative extended Kalman filter (IEKF). Tightly-coupled integration ensures that the integrated system is reliable, even if few known feature points (*i.e.*, less than three) are observed in the images. A new global observability analysis of this tightly-coupled integration is presented to guarantee that the system is observable under the necessary conditions. The analysis conclusions were verified by simulations and field tests. The field tests also indicate that high-precision position (centimeter-level) and attitude (half-degree-level)-integrated solutions can be achieved in a global reference.

## 1. Introduction

High-precision dynamic positioning is in great demand in the applications of automatic driving and intelligent transportation system (ITS). The most popular technology is currently the carrier-phase-based differential GNSS (CDGNSS), which is also known as the real-time kinetic (RTK). It can provide centimeter-level accuracy for real-time applications [1]. However, the stability of CDGNSS relies on the availability of GNSS signals and the correction information sent from the GNSS base station. The CDGNSS cannot operate in urban environments where satellite signals are blocked or in indoor locations where the signals are unavailable.

Image-aided inertial navigation can be an alternative for this satellite-based positioning technology. Imaging sensors (*i.e.*, cameras) do not suffer from satellite signal blocking, are typically low cost and yield effective measurements (*i.e.*, feature points), especially in urban environments. The image measurements captured by the camera can calibrate and confine the time-varying inertial errors. Meanwhile, the positioning of the images can also yield high precision when applied in a close-range area [2,3]. In this paper, a high-precision image-aided inertial navigation technology with close-range features deployed in the surrounding environment is proposed.

Image-based navigation, which is also called vision-based navigation, can be divided into relative and absolute image-based navigation systems [4,5]. In a relative way, features between consecutive image frames are detected and matched to reconstruct the relative changes in position and attitude of the camera. The typical systems of this type include SLAM (simultaneous location and mapping) and visual odometry. After fusing with inertial sensors, the image-aided inertial navigation system is able to cover the limitations and deficiencies of a standalone system [6–9]. Features in the images are matched with absolute features in the real world whose coordinates are known in the navigation environment [2,3,10]. Absolute image-based navigation was used to aid the inertial navigation system (INS) in this study.

In general, there are two architectures for vision and INS integration, which is similar to the architectures of GNSS/INS integration [11], including loosely-coupled and tightly-coupled models [12]. As for the loosely-coupled method, the position and attitude obtained by the camera are used to calibrate the INS errors. Lemay (2011) proposed a loosely-coupled INS/GPS/camera-integrated navigation system using the direct linear transformation (DLT) method to calculate the camera position and attitude [13]. Then, the covariance of the camera position and attitude as a function of pixel noise was analyzed. Similarly, Chu (2012) used the rotation and translation of a camera between adjacent images to aid INS and the rotation, and translation were retrieved from feature matching of the images [14].

In the tightly-coupled strategy, the raw pixel coordinates of feature locations in an image instead of the position and attitude solved from them are fused with the inertial measurements. Specifically, residuals between the detected and the predicted feature locations are utilized to form the measurement update equations in the Kalman filter. Chu (2011) compared the performance of tight and loose camera/IMU integration by simulation, which demonstrated that tight integration yielded a more accurate solution than loose integration. However, tight integration tended to diverge easily [15]. Using a tight method, Vu (2012) proposed a real-time computer vision/GPS/IMU-integrated navigation system. A color camera was utilized to detect traffic lights that had been surveyed in advance. Aided by a camera, the integrated system can maintain the position accuracy at the lane-level in poor GPS environments [10]. Additionally, some relative image-aided inertial navigation systems also use the tightly-coupled architecture [6,8,9,16].

In this study, a monocular camera is integrated with the inertial sensors in a tightly-coupled way. The feature points with known positions are deployed around the navigation area. To obtain high-precision location solutions, the camera is set close to the features. Limited by the field view of the camera, the camera can only observe one or two features in an image most of the time. In this case, tight integration is superior to loose integration, because loose integration cannot solve the position and attitude of the camera when less than three features appear in an image [12]. Then, loose integration fails to aid the inertial sensors and leads to the growing error of the INS.

The Earth-centered Earth-fixed (ECEF) frame is chosen as the global reference coordinate system to derive both the inertial navigation model and the image-based positioning model. The image measurements actually can only provide the bearing information, which is the line-of-sight (LOS) observation for a typical feature. Because the LOS observations are expressed in the Cartesian coordinate system, it is straightforward and effective to fuse it with the inertial states that are also expressed in a Cartesian coordinate system, such as the ECEF frame.

To estimate the optimal states of the image-aided inertial navigation, the iterated EKF (IEKF) method is employed in this paper. Generally, EKF is preferred as the standard method to solve such problems [12]. It was found that the linearized nonlinear model of the camera suffered from divergence when using EKF [15]. However, IEKF reduces linearizing error [17] and has been shown to perform better than EKF in tight integration [15,18]. To combine the inertial data and image data effectively, IEKF is implemented in this study.

The global observability of the image-aided inertial navigation system is analyzed to ensure the effectiveness in fusing these two sensors in a tightly-coupled way. Compared with the local observability, which analyzes the ability to distinguish the states from their neighbors in a small time interval or instantaneously, the global concept describes the ability to estimate the states in the entire time span. Sufficient conditions for the global observability of some integrated systems were presented [19–22]. A brief overview of the related literature is given in Section 3. The global observability analysis approach is not only straightforward and comprehensive, but also provides us with new insights that were unattainable by conventional methods of observability analysis. Covariance simulations and a field test are performed to confirm the theoretical analysis results.

This paper is organized as follows: Section 2 gives the INS and camera models. Section 3 performs the observability analysis of the tightly-coupled camera/IMU integration from a global perspective with a brief review of the related literature. Section 4 describes the mathematical models of the Kalman filter, including the INS model and camera measurement model. Section 5 presents the results of the simulation and field tests using the proposed tightly-coupled algorithm, and Section 6 presents the conclusions.

## 2. Sensor Modeling

### 2.1. INS Modeling

The ECEF frame is taken as the reference frame for the inertial navigation. It is denoted by the *e*-frame. The body frame is defined at the IMU's center, denoted by the *b*-frame with the axes pointing forward, right and down, respectively; the inertial frame is denoted by the *i*-frame; and the local level navigation frame is denoted by the *n*-frame with the axes pointing to north, east and down (NED), respectively.

The dynamic equations for a strapdown INS are given by [11]:

(1) $$\begin{array}{ll} {\dot{r}}_{eb}^{e} & =v_{eb}^{e}\\ {\dot{v}}_{eb}^{e} & =C_{b}^{e}(f^{b}-b_{a})-2\omega_{ie}^{e}\times v_{eb}^{e}+g^{e}\\ {\dot{C}}_{b}^{e} & =C_{b}^{e}(\omega_{eb}^{b}\times )\\ \omega_{eb}^{b} & =\omega_{ib}^{b}-b_{g}-C_{e}^{b}\omega_{ie}^{e}\\ g^{e} & =C_{n}^{e}g^{n} \end{array}$$

where $r_{eb}^{e}$ and $v_{eb}^{e}$ are the position and velocity of the body frame (*i.e.*, located at the IMU center) with respect to the *e*-frame and expressed in the *e*-frame, respectively; $C_{b}^{e}$ is the body attitude matrix with respect to the *e*-frame; *f^b^* is the specific force measured by accelerometers expressed in the *b*-frame; $\omega_{ie}^{e}$ is the Earth's rotation rate expressed in the *e*-frame; *g^e^* is the gravity vector in the *e*-frame; $\omega_{ib}^{b}$ is the body angular rate measured by gyroscopes expressed in the *b*-frame; $\omega_{eb}^{b}$ is the body angular rate with respect to the *e*-frame and expressed in the *b*-frame; ( $\omega_{eb}^{b}\times$) is the skew symmetric matrix of $\omega_{eb}^{b}$; and *b_a_* and *b_g_* are the accelerometer bias and the gyroscope drift, respectively.

### 2.2. Camera Modeling

**Figure 1.**
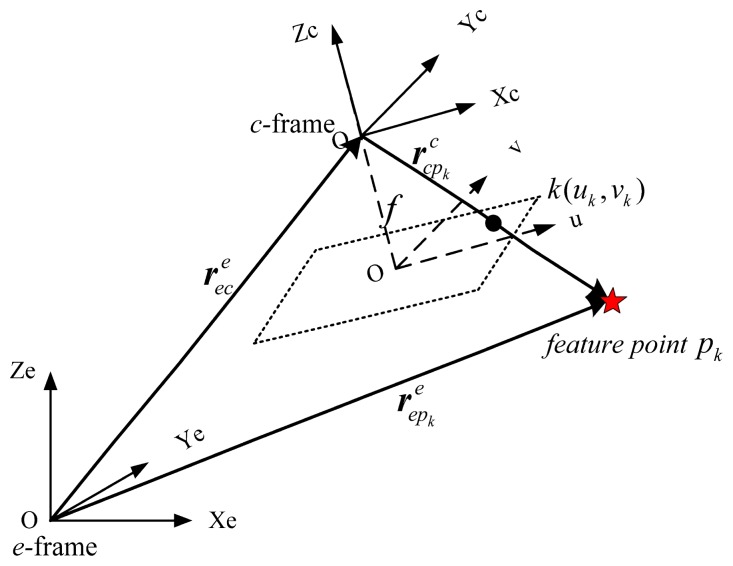
Camera projective model.

An ideal projective (pinhole) camera model was used in this study [23]. The camera frame is defined at the camera's perspective center, denoted by the *c*-frame with the Zc axis along the principal axis and orthogonal to the image plane. The line-of-sight vector $r_{cp_{k}}^{c}$ from the camera to a feature point *p_k_* can be expressed in terms of the position and orientation of the camera and the known position of the feature point (Figure 1).

Mathematically, this can be expressed as follows:

(2) $$r_{cp_{k}}^{c}=C_{e}^{c}(r_{ep_{k}}^{e}-r_{ec}^{e})$$

Obviously, the line-of-sight vector $r_{cp_{k}}^{c}$ and the vector that measures from the image are collinear. Expressing them as scalars yields:

(3) $$\left[\begin{array}{c} r_{cp_{k},x}^{c}\\ r_{cp_{k},y}^{c}\\ r_{cp_{k},z}^{c} \end{array}\right]=\lambda \left[\begin{array}{c} u_{k}-u_{0}\\ v_{k}-v_{0}\\ -f \end{array}\right]$$

The measurement equations for a pinhole camera model can be given by:

(4) $$z=\;\left[\begin{array}{c} u_{k}\\ v_{k} \end{array}\right]+\left[\begin{array}{c} \eta_{u_{k}}\\ \eta_{v_{k}} \end{array}\right]=\left[\begin{array}{c} -f\frac{r_{cp_{k},x}^{c}}{r_{cp_{k},z}^{c}}+u_{0}\\ -f\frac{r_{cp_{k},y}^{c}}{r_{cp_{k},z}^{c}}+v_{0} \end{array}\right]+\left[\begin{array}{c} \eta_{u_{k}}\\ \eta_{v_{k}} \end{array}\right]$$

where $C_{e}^{c}$ is the rotation matrix from the *e*-frame to the *c*-frame. $r_{cp_{k}}^{c}$ denotes the position of the *k*-th feature point expressed in the *c*-frame; $r_{ec}^{e}$ represents the position of the camera center expressed in the e-frame; λ is the unknown scale factor between the collinear vectors; *k*(*u_k_,v_k_*) represents the pixel coordinates of the k-th feature point projected onto the image plane; (*u*_0_,*v*_0_) and *f* are the principal point and the focal length, respectively; and [*η_uk_*,*η_uk_*]*^T^* is the measurement noise vector with covariance $R_{k}=\sigma_{k}^{2}I_{2}$.

Camera intrinsic and distortion parameters can be calibrated using the method given in [24]. This method utilizes a planar checkerboard pattern of known dimensions to calibrate the focal length measured in pixels and the four distortion coefficients. After the camera calibration, the images captured by the camera could be rectified to remove lens distortions.

### 2.3. Camera-IMU Calibration

**Figure 2.**
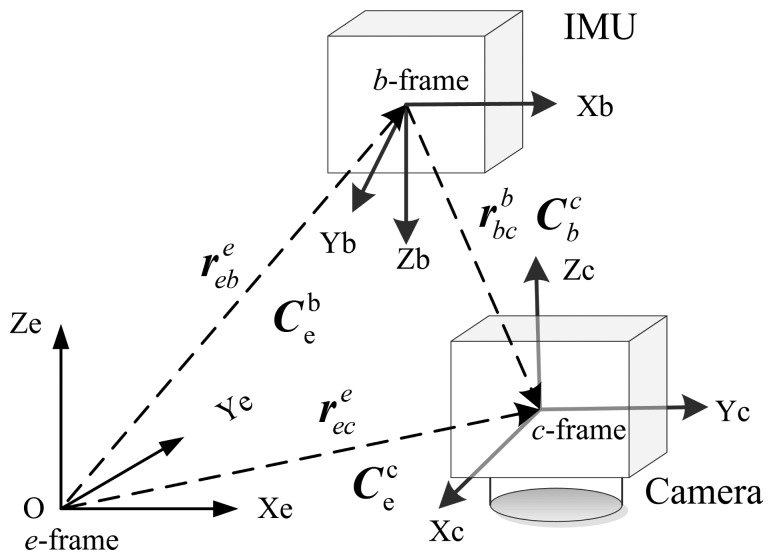
Transformations between the camera and IMU.

To fuse camera observations and inertial measurements effectively, the six-degrees-of-freedom (6-DOF) transformation between the camera and the IMU must be precisely determined. Biased transformation parameters will reduce the accuracy of the estimation process or even lead to divergence in the estimator [18]. As shown in Figure 2, the relative pose (*i.e.*, position and attitude) $r_{bc}^{b}$, $C_{b}^{c}$ between the *c*-frame and the *b*-frame are the transformation parameters requiring calibration. Mirzaei and Roumeliotis (2008) proposed an EKF-based method to compute the relative pose between the camera and the IMU [18]. This approach requires known corner points that are co-planar to be viewable by the camera. Kelly (2010) presented an improved calibration method that can operate without any additional equipment or prior knowledge about the environment [25]. The former method was selected for this study, because sufficient known feature points exist in the environment studied.

## 3. Global Observability Analysis

To integrate the camera and the IMU measurements, the relevant system states must be observable. Observability describes the ability of estimating the states of a system [26]. A system is observable if its state at a certain time can be uniquely determined given a finite sequence of its outputs [19]. Intuitively, this means that the measurements of an observable system provide sufficient information for estimating its state; the observability analysis is necessary, because observability determines the existence of solutions. It is important to understand how the existence of the camera-IMU localization problem depends on the number of observed feature points, their layout and the number of images.

The observability of a camera-IMU-integrated system has recently been studied. The observability properties of a camera-IMU extrinsic calibration (*i.e.*, the estimation of the relative pose of these sensors) have been studied using Lie derivatives [18,25]. However, these approaches are loosely-coupled (*i.e.*, methods that process the IMU and image measurements separately). For instance, these methods first process the poses of the camera and subsequently fuse these with the inertial measurements. This loose method cannot analyze the observability of a tightly-coupled camera-IMU integration, especially when only one or two features are observed in an image.

In a tightly-coupled way, Martinllie (2011) fused the raw data of image measurements with inertial measurements using the concept of continuous symmetries to show that the IMU biases, velocity in the initial body frame and roll and pitch angles are observable for vision-aided INS [27]. In this case, the position of a single feature point located at the origin of the local reference system was known. Similar system observability was investigated in a tightly-coupled way [8,9]. They both focused on how to improve the consistency of the linearized estimator. Hesch (2014) also provided a new method based on factorizing the observability matrix to analytically determine the observability properties of the nonlinear vision-aided INS model [9].

Moreover, the observability rank condition based on Lie derivatives was first proposed to study the observability properties of nonlinear systems [28], and the local observability of the system of interest was investigated. This approach involves complex and cumbersome matrix rank computation. To analyze the observability of nonlinear systems in a global perspective, global observability analysis had been used to examine the observability of nonlinear INS and odometer self-calibration [20,21], the INS/GPS-integrated system [19] and strapdown INS alignment [22], yielding new, comprehensive insights. A global observability analysis can provide extensive instructions regarding the feasibility of estimation under a given condition. In particular, these conditions consider the trajectory, the number of feature points and their layout, as well as the number of monocular images when the same feature points or different feature points are observed. Martinllie (2014) investigated the resolvability of a structure from a motion problem using inertial and visual observations in a closed-form solution [29]. This resolvability analysis basically shows the global observability of a system when only one known feature point is observed. Motivated by this research, the authors extend the global observability analysis to conditions in which more known feature points can be observed and with a different feature point layout.

We study the global observability of the nonlinear system described in Equations (1)–(4) from the observability definition directly [19].

Definition 1: A system is observable if, given the input and output over the finite-time interval, [*t*_0_,*t*], it is possible to uniquely determine the initial state *x*(*t*_0_). Otherwise, the system is unobservable.

For the image-aided inertial navigation system under investigation, the states to be estimated include the position, velocity, attitude, gyro drift and accelerometer bias. Considering the INS alone, the time length is relatively short in the image-aided INS system (less than 1 s or a maximum of a couple of seconds), and the impact of gyro drift is minor in the INS solution [30]. On the other hand, gyro drift produces nonlinear coefficients that make the observability analysis cumbersome and complex. To facilitate the analysis, gyro drift is not considered in the following derivation. The input and output information of the system available includes the specific force measured by accelerometers, the body angular rate measured by gyros and the pixel position of the feature points measured from the images. According to the definition, if the initial states can be uniquely solved given the measurements in a finite-time interval, then the system is proven to be observable.

It is assumed that the platform runs near the Earth's surface at a low speed, so that the Coriolis term, which is $2\omega_{ie}^{e}\;\times \;v_{eb}^{e}$ in Equation (1), can be neglected. Integrating the velocity differential equation over time, the position of the platform at any time *t*∈[*t*_0_,*t*] satisfies the equation:

(5) $$r_{eb}^{e}(t)=r_{eb}^{e}(t_{0})+v_{eb}^{e}(t_{0})\Delta t+\int_{t_{0}}^{t}\int_{t_{0}}^{\tau }a^{e}(\xi )d\xi d\tau$$

This can be simplified into a single integral by integrating the double integral by parts:

(6) $$r_{eb}^{e}(t)=r_{eb}^{e}(t_{0})+v_{eb}^{e}(t_{0})\Delta t+\int_{t_{0}}^{t}\left(t-\tau )a^{e}(\tau \right)d\tau$$

where Δt=*t*–*t*_0_, *a*^e^(*t*) is the platform acceleration expressed in the *e*-frame with the following relationship:

(7) $$a^{e}(t)=C_{b}^{e}(t)(f^{b}(t)-B)+g^{e}$$

where *f^b^*(*t*) is the accelerometer measurement corrupted by the sensor bias *B*. During a short time interval, the bias *B* is a constant term. In a small local area, the gravity vector *g^e^* can also be considered as a constant term.

The attitude changes of $C_{b}^{e}(t)$ caused by Earth's rotation rate are negligible. As will be shown in the next section, a few observed images allow us to determine the observable modes. Additionally, the time scale is only a few seconds. Therefore, during this time, the effects of the Earth's rotation are negligible.

The attitude transform matrix $C_{b}^{e}(t)$ can be rewritten as follows:

(8) $$C_{b}^{e}(t)=C_{b_{0}}^{e}C_{b}^{b_{0}}(t)$$

where $C_{b_{0}}^{e}$ is the initial attitude matrix at time *t*_0_, and $C_{b}^{b_{0}}(t)$ denotes the rotation matrix that rotates the *b*-frame from time *t* to *t*_0_, which can be computed from the outputs of the gyroscopes.

Combining the Equations (6)–(8) yields:

(9) $$r_{eb}^{e}(t)=r_{eb}^{e}(t_{0})+v_{eb}^{e}(t_{0})\Delta t+\frac{1}{2}g^{e}\Delta t^{2}+C_{b_{0}}^{e}S(t)-C_{b_{0}}^{e}\Gamma (t)B$$

where:

(10) $$\begin{array}{ll} S(t) & =\int_{t_{0}}^{t}\left(t-\tau \right)C_{b}^{b_{0}}(t)f^{b}(t)d\tau \\ \Gamma (t) & =\int_{t_{0}}^{t}\left(t-\tau \right)C_{b}^{b_{0}}(t)d\tau \end{array}$$

The terms *S*(*t*) and Γ(*t*) depend only on the measurement of the gyroscopes and accelerometers, both of which can be obtained by integrating the data provided by the gyroscopes and accelerometers delivered during the interval [*t*_0_,*t*].

Because the camera-IMU extrinsic calibration parameters had already been calibrated, it is assumed that the *c*-frame coincided with the *b*-frame. The image measurement of the *k*-th feature point at time *t* actually provided a unitary vector $\mu_{bp_{k}}^{b}(t)$, which can be derived from the left vector in Equation (3). The line-of-sight vector $r_{bp_{k}}^{b}(t)$ expressed in the *b*-frame at time *t* can be written as follows:

(11) $$r_{bp_{k}}^{b}(t)=\lambda_{k}^{t}\mu_{bp_{k}}^{b}(t)$$

where $\lambda_{k}^{t}$ is the unknown scale factor for the k-th feature point observed at time *t*.

Combining $r_{bp_{k}}^{b}=C_{e}^{b}(r_{ep_{k}}^{e}-r_{eb}^{e})$ and (9) yields:

(12) $$\lambda_{k}^{t}\mu_{k}^{t}-p_{k}+r^{b_{0}}-v^{b_{0}}\Delta t-\frac{1}{2}g^{b_{0}}\Delta t^{2}+\Gamma (t)B=S(t)$$

For the sake of simplicity, the following notation is adopted:

(13) $$\begin{array}{ll} p_{k} & =C_{e}^{b_{0}}r_{ep_{k}}^{e}\\ r^{b_{0}} & =C_{e}^{b_{0}}r_{eb_{0}}^{e}\\ v^{b_{0}} & =C_{e}^{b_{0}}v_{eb_{0}}^{e}\\ g^{b_{0}} & =C_{e}^{b_{0}}g^{e}\\ \mu_{k}^{t} & =C_{b}^{b_{0}}(t)\mu_{bp_{k}}^{b}(t) \end{array}$$

where *p_k_* denotes the position of the k-th feature point relative to the *e*-frame expressed in the initial *b*-frame at time *t*_0_; and *r*^*b*^0^^, *v*^*b*^0^^ and *g*^*b*^0^^ denote the initial position, velocity and gravity of the platform relative to the *e*-frame expressed in the initial *b*-frame at time *t*_0_, respectively.

The observability of the image-aided inertial navigation system will be analyzed in two situations. The first situation considers that the same feature points are observed continuously (*i.e.*, the camera constantly tracked some feature points in a particular time period). The second situation considers that different feature points appeared in each image. In both situations, the minimum number of known feature points that appears in an image will be determined, as well as the minimum number of images that should be recorded to make the navigation states observable.

### 3.1. Same Feature Points

#### 3.1.1. One Feature Point

Theorem 1: If only one feature point can be observed continuously, the position, velocity, attitude and accelerometer biases of the camera-IMU-integrated system will be unobservable. Conversely, if the line-of-sight vector changes in the *b*-frame (*i.e.*, the location of the feature point changes on the image plane), the parameters expressed in the initial *b*-frame (*i.e.*, *b*-frame at time *t*_0_), including the position of the feature point and the velocity of the system, are observable. At the same time, the gravity expressed in the initial *b*-frame and the accelerometer bias are observable when the system rotates about at least two axes of the *b*-frame.

Proof: When one feature point is observed continuously, the integrated system provides the Equation (*cf.* (12)):

(14) $$\lambda_{1}^{t}\mu_{1}^{t}-p_{1}+r^{b_{0}}-v^{b_{0}}\Delta t-\frac{1}{2}g^{b_{0}}\Delta t^{2}+\Gamma (t)B=S(t)$$

With time t∈[*t*_1_,*t*_2_,…*t*_n_] increasing, the equations can be stacked and written in matrix *NX*=*L*:

(15) $$\begin{array}{ll} L & ={\left[\begin{array}{cccc} S{\left(t_{1}\right)}^{T}, & S{\left(t_{2}\right)}^{T}, & \cdots , & S{\left(t_{n}\right)}^{T} \end{array}\right]}^{T}\\ X & ={\left[\lambda_{1}^{t_{1}},\ldots ,\lambda_{1}^{t_{n}},{\left(r^{b_{0}}-p_{1}\right)}^{T},{\left(v^{b_{0}}\right)}^{T},{\left(g^{b_{0}}\right)}^{T},B^{T}\right]}^{T}\\ N & =\left[\begin{array}{cccccccc} \mu_{1}^{t_{1}} & 0_{3} & \cdots & 0_{3} & I_{3\times 3} & -\Delta t_{1}I_{3\times 3} & -\frac{1}{2}{\Delta t_{1}}^{2}I_{3\times 3} & \Gamma (t_{1})\\ 0_{3} & \mu_{1}^{t_{2}} & \cdots & 0_{3} & I_{3\times 3} & -\Delta t_{2}I_{3\times 3} & -\frac{1}{2}{\Delta t_{2}}^{2}I_{3\times 3} & \Gamma (t_{2})\\ \vdots & \vdots & \ddots & \vdots & \vdots & \vdots & \vdots & \vdots \\ 0_{3} & 0_{3} & \cdots & \mu_{1}^{t_{n}} & I_{3\times 3} & -\Delta t_{n}I_{3\times 3} & -\frac{1}{2}{\Delta t_{n}}^{2}I_{3\times 3} & \Gamma (t_{n}) \end{array}\right] \end{array}$$

where *n* is the number of images observed, *X* is the vector of unknowns, *L* is the vector integrated from the sensor output and matrix *N* is the coefficient matrix.

The above linear system contains the complete sensor information. If this linear system has a unique solution, the unique solution will be the observable modes; if the matrix *N* has column full rank, the unknowns will be the observable modes.

To analyze the structure of matrix *N*, the column is found to be rank-defective when the unitary vectors $\mu_{1}^{t_{1}}$, $\mu_{1}^{t_{2}}$, ⋯, $\mu_{1}^{t_{n}}$ are collinear. Linearly combining the column vectors [*I*_3×3_, *I*_3×3_, ⋯, *I*_3×3_]^*T*^ can produce a new vector, such as ${\left[\begin{array}{cccc} {\left(\mu_{\begin{array}{cc} 1 & 3\times 1 \end{array}}^{t_{1}}\right)}^{T}, & {\left(\mu_{\begin{array}{cc} 1 & 3\times 1 \end{array}}^{t_{1}}\right)}^{T}, & \cdots , & {\left(\mu_{\begin{array}{cc} 1 & 3\times 1 \end{array}}^{t_{1}}\right)}^{T} \end{array}\right]}^{T}$. If the vectors $\mu_{1}^{t_{1}}$, $\mu_{1}^{t_{2}}$, ⋯, $\mu_{1}^{t_{n}}$ are collinear, linearly combining the first *n* columns of matrix *N* with this new vector will produce the zero vector. The matrix *N* is thus column rank-defective (*i.e.*, a linear combination of column vectors does not change the rank of the matrix). In addition, $\mu_{1}^{t_{1}}$, $\mu_{1}^{t_{2}}$, ⋯, $\mu_{1}^{t_{n}}$ are the line-of-sight vectors projected in the initial *b*-frame; however, these unitary vectors will be collinear only if the location of the feature point is unchanged on the image plane.

Additionally, the matrix *N* is also column rank-defective when the camera-IMU integrated system rotates about fewer than two axes of the *b*-frame. First of all, if the integrated system does not rotate about any axes of the *b*-frame (*i.e.*, it moves in a straight line), $C_{b}^{b_{0}}(t)$ will become the identity matrix *I*_3×3_ and the last columns of ${\left[\begin{array}{cccc} \Gamma {\left(t_{1}\right)}^{T}, & \Gamma {\left(t_{2}\right)}^{T}, & \cdots , & \Gamma {\left(t_{n}\right)}^{T} \end{array}\right]}^{T}$ will become (*cf.* (10)):

$${\left[\begin{array}{cccc} {\left(\frac{1}{2}{\Delta t_{1}}^{2}I_{3\times 3}\right)}^{T}, & {\left(\frac{1}{2}{\Delta t_{2}}^{2}I_{3\times 3}\right)}^{T}, & \cdots , & {\left(\frac{1}{2}{\Delta t_{n}}^{2}I_{3\times 3}\right)}^{T} \end{array}\right]}^{T}$$

The coefficient matrix of *g*^*b*^0^^ and *B* will become linearly correlated, which leads to the matrix *N* being column rank-defective. It is actually that the quantities *g*^*b*^0^^ and *B* are not separable. Secondly, if the integrated system rotates about one axes of the *b*-frame, it can be assumed that the system rotates about the vertical axe, which is expected for horizontal movements. This case leads to the matrix $C_{b}^{b_{0}}(t)$ with a structure like:

$$\left[\begin{array}{ccc} a(t) & b(t) & 0\\ c(t) & d(t) & 0\\ 0 & 0 & 1 \end{array}\right]$$

Then, the third column of ${\left[\begin{array}{cccc} \Gamma {\left(t_{1}\right)}^{T}, & \Gamma {\left(t_{2}\right)}^{T}, & \cdots , & \Gamma {\left(t_{n}\right)}^{T} \end{array}\right]}^{T}$ will become (*cf.* (10)):

$${\left[\begin{array}{cccc} {\left(0,\;\;0,\;\;\frac{1}{2}{\Delta t_{1}}^{2}\right)}^{T}, & {\left(0,\;0,\;\frac{1}{2}{\Delta t_{2}}^{2}\right)}^{T}, & \cdots , & {\left(0,\;0,\;\frac{1}{2}{\Delta t_{n}}^{2}\right)}^{T} \end{array}\right]}^{T}$$

This vector is linearly correlated with the third column of:

$${\left[\begin{array}{cccc} {\left(-\frac{1}{2}{\Delta t_{1}}^{2}I_{3\times 3}\right)}^{T}, & {\left(-\frac{1}{2}{\Delta t_{2}}^{2}I_{3\times 3}\right)}^{T}, & \cdots , & {\left(-\frac{1}{2}{\Delta t_{n}}^{2}I_{3\times 3}\right)}^{T} \end{array}\right]}^{T}$$

Thus, matrix *N* is column rank-defective. Actually, the third component of *g*^*b*^0^^ is not separable from the third component of *B* (*i.e.*, vertical accelerometer bias), and they are unobservable.

Similarly, when the integrated system rotates about one of the other axes of the *b*-frame, the accelerometer bias of the rotational axis will be unobservable. It can also be concluded that rotating about at least two axes of the *b*-frame will ensure the quantities *g*^*b*^0^^ and *B* are separable, as the structure of matrix $C_{b}^{b_{0}}(t)$ is different with increasing time.

The matrix *N* is full rank if the vectors $\mu_{1}^{t_{1}}$, $\mu_{1}^{t_{2}}$, ⋯, $\mu_{1}^{t_{n}}$ are non-collinear, and the camera-IMU system rotates about at least two axes of the *b*-frame. Under these conditions, the linear system has a unique solution *X* and all unknowns are observable. The observable modes are the parameters projected in the initial *b*-frame, including $C_{e}^{b_{0}}(r_{eb_{0}}^{e}-r_{ep_{1}}^{e})$, *v*^*b*^0^^, *g*^*b*^0^^ and *B*. When the system rotates about less than two axes of the *b*-frame, the latter condition is not met. In this case, *g*^*b*^0^^ and *B* are inseparable. However, these two quantities can be combined into one quantity to ensure that the new coefficient matrix is full rank. Hence, the observable modes are $C_{e}^{b_{0}}(r_{eb_{0}}^{e}-r_{ep_{1}}^{e})$ and *v*^*b*^0^^. The unobservable modes are *g*^*b*^0^^ and *B*. Furthermore, this conclusion is similar to the observability conclusion given by [27], which used the concept of continuous symmetries.

If the trajectory of the perspective center of the camera and the location of the feature point are coplanar, all of the vectors $\mu_{1}^{t_{1}}$, $\mu_{1}^{t_{2}}$, ⋯, $\mu_{1}^{t_{n}}$ will belong in the same plane. This means that these vectors can be projected to a frame in which all of them have the last component equal to zero. In the new frame, the linear system *NX*=*L* can be divided into two parts: one part corresponds to the first two lines of (14) for *t*∈[*t*_1_,*t*_2_,…*t_n_*]; the other part corresponds to the third line of (14) for *t*∈[*t*_1_,*t*_2_,…*t_n_*], which only involves the third component of *_v_^b^*^0^, *g*^*b*^0^^ and *B* expressed in the new frame. Matrices *N*_1_ and *N*_2_ represent the two parts of the linear system; the size of *N*_1_ is 2*n*×(n+8), and the size of *N*_2_ is *n*×4. For the case without accelerometer bias, the size of *N*_1_ becomes 2*n*×(n+6) and the size of *N*_2_ becomes *n*×3. To ensure the system has a unique solution, *n* should be at least nine for the biased case and at least seven for the unbiased case.

Conversely, if the trajectory of the perspective center of the camera and the location of the feature point spans the 3D space, the size of matrix *N* is 3*n*×(*n*+10) for the biased case and 3*n*×(*n*+9) for the unbiased case, because the last three columns disappear when there is no accelerometer bias. To ensure the matrix *N* has column full rank, *n* should be at least six for the biased case and at least five for the unbiased case.

The results of this subsection are summarized with the following properties:

Property 1: In the planar case, to estimate the observable modes given in Theorem 1, the minimum number of camera images is eight, with the assumption of accelerometer bias (*i.e.*, the observability requires at least eight images from eight distinct camera poses). For the case without accelerometer bias, the minimum number of camera images becomes six.

Property 2: For the 3D case, to estimate the observable modes given in Theorem 1, the minimum number of camera images is six, with the assumption of accelerometer bias. For the case without accelerometer bias, the minimum number of camera images becomes five.

It can be observed that under the observable conditions given in Theorem 1, the coplanar case has the same observable properties as the 3D case, but more images are required than in the 3D case to solve the observable modes. Therefore, in the following analysis, only the position of the perspective center of the camera and the feature points spanning the 3D space are considered.

#### 3.1.2. Two Feature Points

Theorem 2: If two feature points can be observed continuously and the following conditions are met:

(1) The feature points are not in a vertical line.
(2) Both line-of-sight vectors for the two features change in the *b*-frame (*i.e.*, the locations of the feature points change on the image plane).
(3) The system rotates about at least two axes of the *b*-frame.

then the position, velocity, attitude and the accelerometer biases of the camera-IMU integrated system are observable.

Proof: When two known feature points are observed continuously, the integrated system provides the equations:

(16) $$\begin{array}{cc} \lambda_{1}^{t}\mu_{1}^{t}-p_{1}+r^{b_{0}}-v^{b_{0}}\Delta t-\frac{1}{2}g^{b_{0}}\Delta t^{2}+\Gamma (t)B & =S(t)\\ \lambda_{2}^{t}\mu_{2}^{t}-p_{2}+r^{b_{0}}-v^{b_{0}}\Delta t-\frac{1}{2}g^{b_{0}}\Delta t^{2}+\Gamma (t)B & =S(t) \end{array}$$

With the time *t*∈[*t*_1_,*t*_2_,…*t_n_*] increasing, the equations can be stacked and written in matrix *NX*=*L*:

(17) $$\begin{array}{ll} L & ={\left[\begin{array}{cccc} S{\left(t_{1}\right)}^{T}, & S{\left(t_{2}\right)}^{T}, & \cdots , & S{\left(t_{n}\right)}^{T},\;\;\;\begin{array}{cccc} S{\left(t_{1}\right)}^{T}, & S{\left(t_{2}\right)}^{T}, & \cdots , & S{\left(t_{n}\right)}^{T} \end{array} \end{array}\right]}^{T}\\ X & ={\left[\lambda_{1}^{t_{1}},\ldots ,\lambda_{1}^{t_{n}},\lambda_{2}^{t_{1}},\ldots ,\lambda_{2}^{t_{n}},{\left(r^{b_{0}}-p_{1}\right)}^{T},{\left(r^{b_{0}}-p_{2}\right)}^{T},{\left(v^{b_{0}}\right)}^{T},{\left(g^{b_{0}}\right)}^{T},B^{T}\right]}^{T}\\ N & =\left[\begin{array}{ccccccccccc} \mu_{1}^{t_{1}} & \cdots & 0_{3} & 0_{3} & \cdots & 0_{3} & I_{3\times 3} & 0_{3} & -\Delta t_{1}I_{3\times 3} & -\frac{1}{2}{\Delta t_{1}}^{2}I_{3\times 3} & \Gamma (t_{1})\\ \vdots & \ddots & \vdots & \vdots & \ddots & \vdots & \vdots & \vdots & \vdots & \vdots & \vdots \\ 0_{3} & \cdots & \mu_{1}^{t_{n}} & 0_{3} & \cdots & 0_{3} & I_{3\times 3} & 0_{3} & -\Delta t_{n}I_{3\times 3} & -\frac{1}{2}{\Delta t_{n}}^{2}I_{3\times 3} & \Gamma (t_{n})\\ 0_{3} & \cdots & 0_{3} & \mu_{2}^{t_{1}} & \cdots & 0_{3} & 0_{3} & I_{3\times 3} & -\Delta t_{1}I_{3\times 3} & -\frac{1}{2}{\Delta t_{1}}^{2}I_{3\times 3} & \Gamma (t_{1})\\ \vdots & \ddots & \vdots & \vdots & \ddots & \vdots & \vdots & \vdots & \vdots & \vdots & \vdots \\ 0_{3} & \cdots & 0_{3} & 0_{3} & \cdots & \mu_{2}^{t_{n}} & 0_{3} & I_{3\times 3} & -\Delta t_{n}I_{3\times 3} & -\frac{1}{2}{{\Delta t_{n}}^{2}}_{3\times 3} & \Gamma (t_{n}) \end{array}\right] \end{array}$$

According to Theorem 1, if the second and third conditions are met, the observable modes for the above system are $\lambda_{1}^{t}$, $\lambda_{2}^{t}$, *r*^*b*^0^^–*p*_1_, *r*^*b*^0^^–*p*_2_, *v*^*b*^0^^, *g*^*b*^0^^ and *B*. Then, the parameter *p*_2_–*p*_1_ is also observable and can be rewritten as:

(18) $$p_{2}-p_{1}=C_{e}^{b_{0}}(r_{ep_{2}}^{e}-r_{ep_{1}}^{e})$$

Furthermore:

(19) $$g^{b_{0}}=C_{e}^{b_{0}}g^{e}$$

The vector $r_{ep_{2}}^{e}-r_{ep_{1}}^{e}$ and the local gravity *g^e^* are all known parameters. Under the assumption of the first condition, they are also linearly independent. The attitude matrix $C_{e}^{b_{0}}$ thus has a unique solution. This is because for any two linearly-independent vectors, if their coordinates in two arbitrary frames are given, then the attitude matrix between the two frames can be uniquely determined [31]. Therefore, the initial attitude matrix $C_{e}^{b_{0}}$ is observable. At the same time, the position and velocity are also observable for the relation $r_{eb}^{e}={\left(C_{e}^{b_{0}}\right)}^{T}r^{b_{0}}$, $v_{eb}^{e}={\left(C_{e}^{b_{0}}\right)}^{T}v^{b_{0}}$.

Because the size of matrix *N* is 6*n*×(2*n*+15) for the biased case and 6*n*×(2*n*+12) for the unbiased case, the last three columns disappear when there is no accelerometer bias. To ensure the matrix *N* has column full rank, *n* should be at least four for the biased case and at least three for the unbiased case. This result is summarized with the following property:

Property 3: To estimate the observable states given in Theorem 2, the minimum number of camera images is four for the case with accelerometer bias. For the case without accelerometer bias, the minimum number of camera images reduces to three.

#### 3.1.3. Three Feature Points and More

Theorem 3: For three or more feature points that can be observed continuously, if all of the feature points are in a straight line, which is not a vertical line, the position, velocity, attitude and accelerometer biases of the camera-IMU integrated system are observable when the following conditions are met:

(1) All of the line-of-sight vectors for the features change in the *b*-frame (*i.e.*, the locations of the feature points change on the image plane).
(2) The system rotates about at least two axes of the *b*-frame.

If the feature points are not in a straight line, then the position, velocity and attitude of the camera-IMU-integrated system are observable. At the same time, the accelerometer bias is observable when the system rotates about at least two axes of the *b*-frame.

Proof: Since observing more than three feature points provides the same observable information as observing only three feature points; observing more feature points only improves the estimated accuracy [32]. Thus, only the case of three observed feature points needs to be considered.

Firstly, if the three feature points are in a straight line that is not vertical, any two of the three feature points will not be in a vertical line. This meets the first condition of Theorem 2. Meanwhile, the first condition and second condition are the same as the second condition and third condition of Theorem 2. Therefore, the system modes are observable.

Secondly, when the three feature points are not in a straight line and their positions are known, the scale factors, such as $\lambda_{1}^{t}$, $\lambda_{2}^{t}$and $\lambda_{3}^{t}$, can be directly determined by space resection [32]. *X* will not contain any unknown scale factors and becomes:

$$X={\left[{\left(r^{b_{0}}-p_{1}\right)}^{T},{\left(r^{b_{0}}-p_{2}\right)}^{T},{\left(r^{b_{0}}-p_{3}\right)}^{T},{\left(v^{b_{0}}\right)}^{T},{\left(g^{b_{0}}\right)}^{T},B^{T}\right]}^{T}$$

According to Theorem 1, if the system rotates about at least two axes of the *b*-frame, all the states of *X* are observable. However, if the system rotates about less than two axes of the *b*-frame, the states of *X* are observable, except for *g*^*b*^0^^ and *B*, which are inseparable. Due to the three feature points not being in a straight line, the vectors $r_{ep_{2}}^{e}-r_{ep_{1}}^{e}$ and $r_{ep_{3}}^{e}-r_{ep_{1}}^{e}$ must be linearly independent. These two vectors can form equations, such as (18). Additionally, the attitude matrix $C_{e}^{b_{0}}$ in Equation (18) can be uniquely determined [31]. Then, using this attitude matrix $C_{e}^{b_{0}}$ to project the observable modes of *X* in the *e*-frame, the observable modes are obtained.

Additionally, the size of matrix *N* is 9*n*×(3*n*+18) for the biased case and 9*n*×(3*n*+15) for the unbiased case. To ensure the matrix *N* has column full rank, *n* should be at least three for the biased case and at least three for the unbiased case. This result is summarized with the following property:

Property 4: To estimate the observable states given in Theorem 3 for the three feature points observed, the minimum number of camera images is three for the case with accelerometer bias. For the case without accelerometer bias, the minimum number of camera images is three.

### 3.2. Different Feature Points

Theorem 4: When different feature points can be observed in each image, if the line-of-sight vectors change in the body frame (*i.e.*, the locations of the feature points on the image plane change), the position, velocity and attitude of the camera-IMU integrated system are observable. At the same time, the accelerometer bias is observable when the system rotates about at least two axes of the *b*-frame.

Proof: Because observing two or more feature points at a time provides more information than the situation with one feature point [32], the case that observing one feature point at a time is observable must be proven. Assuming that each image observes a different known feature point, the model can be described as follows:

(20) $$\lambda_{n}^{t_{n}}\mu_{n}^{t_{n}}-p_{n}+r^{b_{0}}-v^{b_{0}}\Delta t_{n}-\frac{1}{2}g^{b_{0}}{\Delta t_{n}}^{2}+\Gamma (t_{n})B=S(t_{n})$$

The difference between *t_n_* and *t*_1_ in the above equation yields an equation that contains *p_n_*–*p*_1_ and can be rewritten using the initial attitude and locations of feature points:

(21) $$p_{n}-p_{1}=C_{e}^{b_{0}}(r_{ep_{n}}^{e}-r_{ep_{1}}^{e})$$

Because the attitude matrix $C_{e}^{b_{0}}$ can be linearized with small attitude errors *ε* and the approximate attitude matrix ${\tilde{C}}_{e}^{b_{0}}$ [11], which is known and can be provided by the alignment step of inertial navigation, the following can be derived:

(22) $$C_{e}^{b_{0}}=(I-\left[ɛ\times \right]){\tilde{C}}_{e}^{b_{0}}$$

(23) $$p_{n}-p_{1}={\tilde{C}}_{e}^{b_{0}}(r_{ep_{n}}^{e}-r_{ep_{1}}^{e})+\left[{\tilde{C}}_{e}^{b_{0}}(r_{ep_{n}}^{e}-r_{ep_{1}}^{e})\times \right]ɛ$$

where [*ε*×] and $\left[{\tilde{C}}_{e}^{b_{0}}(r_{ep_{n}}^{e}-r_{ep_{1}}^{e})\times \right]$ denote the skew symmetric matrix of the small attitude errors *ε* and the vector ${\tilde{C}}_{e}^{b_{0}}(r_{ep_{n}}^{e}-r_{ep_{1}}^{e})$ respectively.

During the time t∈[*t*_1_,*t*_2_,…*t_n_*], the equations' difference between *t_n_* and *t*_1_ can be stacked and written in matrix *NX*=*L* as:

(24) $$\begin{array}{ll} X & ={\left[\lambda_{1}^{t_{1}},\ldots ,\lambda_{n}^{t_{n}},{ɛ}^{T},{\left(v^{b_{0}}\right)}^{T},{\left(g^{b_{0}}\right)}^{T},B^{T}\right]}^{T}\\ L & ={\left[\begin{array}{cccc} S{\left(t_{2}\right)}^{T}-S{\left(t_{1}\right)}^{T}, & S{\left(t_{3}\right)}^{T}-S{\left(t_{1}\right)}^{T}, & \cdots , & S{\left(t_{n}\right)}^{T}-S{\left(t_{1}\right)}^{T} \end{array}\right]}^{T}\\ N & =\left[\begin{array}{ccccccccc} -\mu_{1}^{t_{1}} & \mu_{2}^{t_{2}} & 0_{3} & \cdots & 0_{3} & \left[{\tilde{C}}_{e}^{b_{0}}(r_{ep_{2}}^{e}-r_{ep_{1}}^{e})\times \right] & -(\Delta t_{2}-\Delta t_{1})I_{3\times 3} & -\frac{1}{2}({\Delta t_{2}}^{2}-{\Delta t_{1}}^{2})I_{3\times 3} & \Gamma (t_{2})-\Gamma (t_{1})\\ -\mu_{1}^{t_{1}} & 0_{3} & \mu_{3}^{t_{3}} & \cdots & 0_{3} & \left[{\tilde{C}}_{e}^{b_{0}}(r_{ep_{3}}^{e}-r_{ep_{1}}^{e})\times \right] & -(\Delta t_{3}-\Delta t_{1})I_{3\times 3} & -\frac{1}{2}({\Delta t_{3}}^{2}-{\Delta t_{1}}^{2})I_{3\times 3} & \Gamma (t_{3})-\Gamma (t_{1})\\ \vdots & \vdots & \vdots & \ddots & \vdots & \vdots & \vdots & \vdots & \vdots \\ -\mu_{1}^{t_{1}} & 0_{3} & 0_{3} & \cdots & \mu_{n}^{t_{n}} & \left[{\tilde{C}}_{e}^{b_{0}}(r_{ep_{n}}^{e}-r_{ep_{1}}^{e})\times \right] & -(\Delta t_{n}-\Delta t_{1})I_{3\times 3} & -\frac{1}{2}({\Delta t_{n}}^{2}-{\Delta t_{1}}^{2})I_{3\times 3} & \Gamma (t_{n})-\Gamma (t_{1}) \end{array}\right] \end{array}$$

Analyzing the structure of matrix *N*, it is found that $\mu_{1}^{t_{1}}$, $\mu_{1}^{t_{2}}$ and $\mu_{1}^{t_{n}}$ must be linearly independent. Otherwise, the matrix is rank defective. Similarly, only the line-of-sight vectors change in the body frame, which means that the locations of the feature points on the image plane change. According to Theorem 1, if the system rotates about at least two axes of the *b*-frame, all of the states of *X* are observable. However, if the system rotates about less than two axes of the *b*-frame, the states of *X* are observable, except for *g*^*b*^0^^ and *B*, which are inseparable. Once the attitude error *ε* is determined, the initial attitude matrix $C_{e}^{b_{0}}$ can also be solved. Then, the estimated parameters are substituted into Equation (20) to obtain *r*^*b*^0^^. Finally, the states $r_{eb}^{e}$ and $v_{eb}^{e}$ in the *e*-frame can also be solved and are found to be observable.

Additionally, the size of matrix *N* is 3(*n*–1)×(*n*+12) for the biased case and is 3(*n*–1)×(*n*+9) for the unbiased case. To ensure the matrix *N* has column full rank, *n* should be at least eight for the biased case and at least six for the unbiased case. This result is summarized with the following property:

Property 5: To estimate the observable states given in Theorem 4, the minimum number of camera images is eight for the case with the accelerometer bias. For the case without accelerometer bias, the minimum number of camera images becomes six.

In summary, to keep the camera-IMU integrated system observable, at least two feature points should be tracked for the situation that the same feature points are observed in the images, as long as the locations of both feature points on the image plane change. When only one feature point is observed at a time in the images, the system will be observable if the observed feature points are different points from image to image and the locations of the feature points on the image plane change. Additionally, if the accelerometer bias needs to be estimated, the system should rotate about at least two axes of the *b*-frame.

## 4. Kalman Filter Implementation

A Kalman filter is a minimum variance estimator and is comprised of a system model and a measurement model [17]. These two models are represented by time update equations and measurement update equations, respectively. In the tightly-coupled camera/IMU approach, the time update equations were derived from INS error models and the measurement update equations were derived from the camera error model. The measurement update restricts IMU error growth and keeps the error bounded.

### 4.1. INS Error Model: System Model

To compute the inertial data collected by the IMU, the INS mechanization algorithm derived in the *e*-frame was implemented in this study. The state estimate is propagated forward in time using the INS equations, and the attitude is updated using a quaternion algorithm [33]. The INS error model can be expressed as follows [11]:

(25) $$\begin{array}{ll} \delta {\dot{r}}_{eb}^{e} & =\delta v_{eb}^{b}\\ \delta {\dot{v}}_{eb}^{b} & =(C_{b}^{e}f^{b})\times \phi -2\omega_{ie}^{e}\times \delta v_{eb}^{b}+\delta g^{e}+C_{b}^{e}b_{a}\\ \dot{\phi } & =-\omega_{ie}^{e}\times \phi -C_{b}^{e}b_{g} \end{array}$$

where *e* represents the navigation frame (*i.e.*, the *e*-frame); $\delta {\dot{r}}_{eb}^{e}$, $\delta {\dot{v}}_{eb}^{e}$ and *φ̇* are the rate of the position error, the velocity error and attitude error expressed in the *e* -frame, respectively; $\omega_{ie}^{e}$ is the angular rate of the *e* -frame relative to the *i*-frame, expressed in the *e*-frame; *δg^e^* is the gravity error in the *e*-frame; *b_g_*, *b_a_* are the inertial sensor errors; and $C_{b}^{e}$ is the rotation matrix from the body frame (*i.e.*, the *b*-frame) to the navigation frame (*i.e.*, the *e*-frame).

Due to the errors in time-varying sensors, a first-order Gauss–Markov process is used to model sensor errors [34]:

(26) $$\dot{b}=-\frac{1}{T}b+w$$

where *b* represent the errors of the inertial sensors, including the gyroscopes' and accelerometer bias error (*b_g_*, *b_a_*); *T* is the correlation time of the first-order Gauss–Markov process; and *w* is the driving white noise.

A 15-state vector for the navigation filter was created, which is represented as follows.

(27) $$x={\left[\begin{array}{ccccc} {\left(\delta r_{eb}^{e}\right)}^{T} & {\left(\delta v_{eb}^{e}\right)}^{T} & {\left(\phi \right)}^{T} & {\left(b_{g}\right)}^{T} & {\left(b_{a}\right)}^{T} \end{array}\right]}^{T}$$

### 4.2. Camera Error Model: Measurement Model

Modeling the tightly-coupled camera measurement equation requires only raw image observations, such as pixel coordinates. To utilize the camera measurement model described in Section 2, Equation (2) must be linearized at the IMU center rather than the camera center. These two reference centers can be transformed in the navigation frame as follows (Figure 2):

(28) $$r_{ec}^{e}=r_{eb}^{e}+C_{b}^{e}r_{bc}^{b}$$

where $r_{eb}^{e}$ and $r_{ec}^{e}$ are the position of the IMU and camera in the *e*-frame, respectively; $r_{bc}^{b}$ is the lever arm between the IMU and camera projective center in the body frame.

Then, Equation (2) can be rewritten as:

(29) $$r_{cp_{k}}^{c}=C_{b}^{c}C_{e}^{b}(r_{ep_{k}}^{e}-r_{ec}^{e})$$

where $C_{b}^{c}$ is the rotation matrix from the body frame to the camera frame, which is known and must be calibrated in advance.

Substituting Equation (28) into Equation (29), new camera measurement equations with relations to the IMU position center $r_{eb}^{e}$ and attitude matrix $C_{b}^{e}$ are obtained as follows.

(30) $$r_{cp_{k}}^{c}=C_{b}^{c}(C_{e}^{b}(r_{ep_{k}}^{e}-r_{eb}^{e})-r_{bc}^{b})$$

Therefore, the linearization can be presented as a two-step process. Firstly, the perturbation of a pixel coordinate *δ_Zk_* is related to the perturbation of the line of sight vector $r_{cp_{k}}^{c}$. According to Equation (4), this relationship can be replaced as follows:

(31) $$\delta z_{k}=H_{1}\delta r_{cp_{k}}^{c}$$

(32) $$H_{1}=\left[\begin{array}{ccc} -\frac{f}{r_{cp_{k},z}^{c}} & 0 & \frac{f\cdot r_{cp_{k},x}^{c}}{{\left(r_{cp_{k},z}^{c}\right)}^{2}}\\ 0 & -\frac{f}{r_{cp_{k},z}^{c}} & \frac{f\cdot r_{cp_{k},y}^{c}}{{\left(r_{cp_{k},z}^{c}\right)}^{2}} \end{array}\right]$$

Secondly, the perturbation of line-of-sight vector $r_{cp_{k}}^{c}$ is related to the position error and attitude error, which are $\delta r_{eb}^{e}$ and *φ*, respectively:

(33) $$\delta r_{cp_{k}}^{c}=H_{2}\left[\begin{array}{c} \delta r_{eb}^{e}\\ \phi \end{array}\right]$$

(34) $$H_{2}=\left[\begin{array}{cc} -C_{b}^{c}C_{n}^{b} & C_{b}^{c}C_{e}^{b}(r_{eb}^{e}\times ) \end{array}\right]$$

Combining Equations (31)–(34), the following is obtained:

(35) $$\delta z_{k}=H_{1}H_{2}\left[\begin{array}{c} \delta r_{eb}^{e}\\ \phi \end{array}\right]$$

This equation will be used as the measurement equation in the tightly-coupled Kalman filter. The size of the measurement vector varies depending on the number of feature points detected.

After modeling the system and measurement models, they can be used in the standard extended Kalman filter equations. However, IEFK requires multiple iterative updates before the state error vector converges to a threshold value [17,18]. EKF only expands the measurement model in a Taylor series around the last optimal estimate state. If expanding the Taylor series around the new estimate at every update, IEKF thus has the benefit of reducing the linearizing error [17]. Furthermore, the camera measurement model is a highly nonlinear function, as described in Equations (2)–(4), which can explain why IEKF performs better than EFK in this tightly-coupled camera/IMU integration.

## 5. Experimental Results

To validate the proposed tightly-coupled algorithm and its observability properties when known feature points are available, simulation experiments and field tests were performed.

### 5.1. Simulation

Three sets of trajectories are simulated to validate the observability properties given in Section 3.

**Figure 3.**
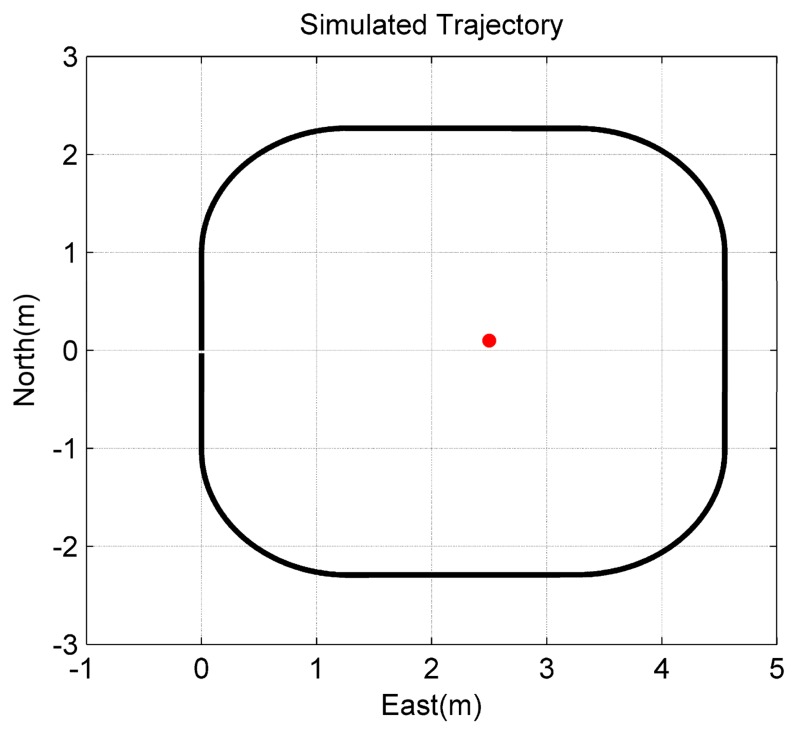
Simulated trajectory of the camera/IMU-integrated platform and the layout of the only feature point.

**Figure 4.**
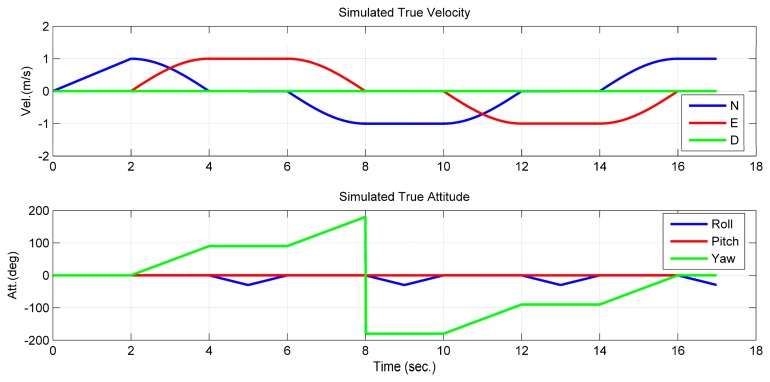
Simulated motions of the camera/IMU-integrated platform. N, E, D, north, east and down, respectively.

To verify the observability conclusion given in Theorem 1, the first simulation assumed that only one feature point can be tracked and observed continuously. The camera/IMU-integrated platform moved in a square trajectory with the camera looking at the center of the square (Figure 3). Additionally, the only feature point was located at the center of the square with a small offset to the northeast. Meanwhile, the platform rotated about the first and the third axes of the *b*-frame, in turn (Figure 4). Additionally, there is no doubt that the location of the feature point was changed constantly on the image plane.

The IMU noise characteristics are the same as the MTi-G IMU used in the real experiments (Table 1). The IMU measurements are sampled at 100 Hz. The parameters of the simulated camera are also the same as the Basler camera in the real-world experiment (Table 1). The image measurements are captured at 10 Hz.

**Figure 5.**
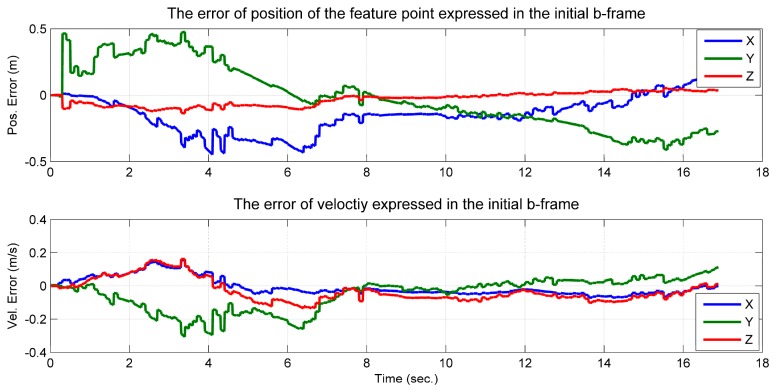
Error of position of the feature point and velocity of the camera/IMU-integrated platform expressed in the initial *b*-frame.

**Figure 6.**
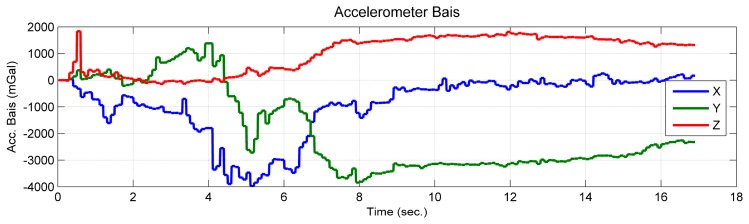
Estimated accelerometer bias of the camera/IMU-integrated system.

**Figure 7.**
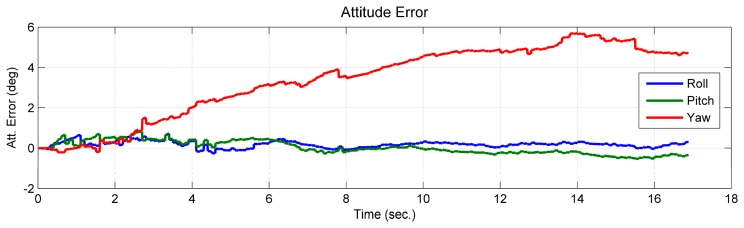
Attitude error of the camera/IMU-integrated platform.

Comparing the estimated value and the simulated true value, it was shown that the position of the feature point and the velocity, which are expressed in the initial *b*-frame, were observable (Figure 5). The curves of the estimated accelerometer biases also indicate that the accelerometer biases were observable (Figure 6). Additionally, the roll and pitch angles were observable (Figure 7). This is because the gravity vector known in the *e*-frame provided the observable information for the roll and pitch angles [27]. According to Equation (19), the gravity vector expressed in the initial *b*-frame was also observable. Therefore, when the conditions in Theorem 1 are met, the quantities given in the conclusion are observable. However, the yaw angle was drifting and unobservable (Figure 7). Here, the unobservability of the yaw angle leads to the velocity and position of the system being unobservable in the *e*-frame, which verified the first part of Theorem 1.

**Figure 8.**
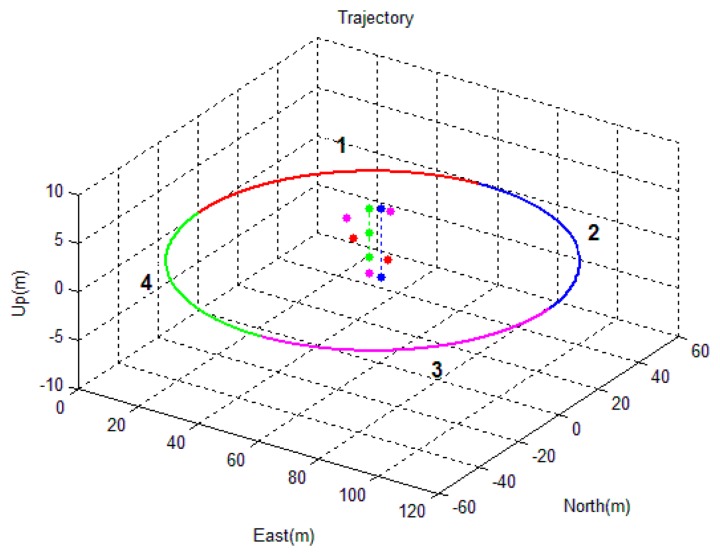
Simulated circle trajectory of the camera/IMU-integrated platform and the layout of the feature points.

The second simulated scenario considers a camera/IMU-integrated platform moving in a circle (Figure 8) with its camera looking at the center of the circle. Some feature points were distributed near the center of the circle, allowing the camera to track the feature points continuously. The trajectory of the circle was divided into four parts, which were indicated by different colors in Figure 8. Part 1 and Part 2 are shown to observe two feature points, while Part 3 and Part 4 are shown to observe three feature points. The difference between parts with the same number of features is that the feature points are located in a vertical line in the case of Parts 2 and 4, while this is not the case for Parts 1 and 3. The performances of the sensors are the same as the sensors simulated in the first simulation.

**Figure 9.**
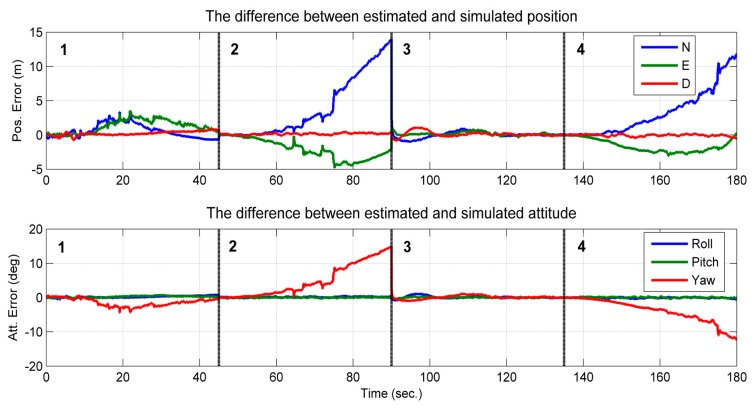
Position and attitude error of the tightly-coupled image/INS solution in the second simulation.

The processed image and the tightly-coupled INS results are shown in Figure 9. The figure shows the position and attitude error of the corresponding trajectory shown in Figure 8. The integrated solution of the position and attitude diverge in the second and fourth section of the trajectory (Figure 9). In these two sections, the feature points are in a vertical line; in the other two sections, the errors are bounded, and a sharp decrease of the error appears at the 90th second (*i.e.*, the time in the middle of the second and third section), when the layout of the feature points changed from two feature points in a vertical line to three feature points that span the 3D space. When the observed feature points are not in a vertical line, the estimated states are thus observable, which confirms the conclusions of Theorem 2 and Theorem 3. Furthermore, it can be observed that the attitude error of the roll and pitch angles are bounded in the entire time span. This is because the gravity vector sensed by the IMU provided the observable information for these two states [27]. Additionally, the observable information for the yaw angle can only be provided by observing the feature points that are not in a vertical line.

**Figure 10.**
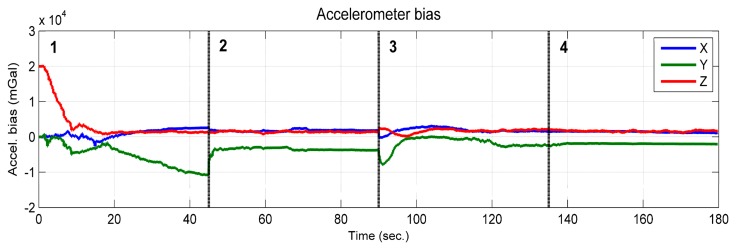
Accelerometer bias.

According to the observability analysis given in Section 3.1.1, the vertical component of the accelerometer bias is observable when the platform rotates about at least two axes of the *b*-frame. However, as was shown in Figure 10, the estimated vertical component of the accelerometer bias (red line) was observable, which converged toward the true value from an intentionally biased initial value, although it only rotated about the vertical axes of the *b*-frame. This is because the sufficient condition “The system rotates about at least two axes of the *b*-frame” is given based on the assumption that the gravity vector expressed in the initial *b*-frame has three degrees of freedom. In reality, the gravity vector expressed in the *e*-frame is known. This leads to the magnitude of the gravity vector being known. Then, the degrees of freedom of the gravity vector expressed in the initial *b*-frame become two [29]. In this case, the observability conditions can be loosened. If considering that the magnitude of the gravity vector is known, the complete theoretical analysis will become very complicated and outside the scope of the paper.

**Figure 11.**
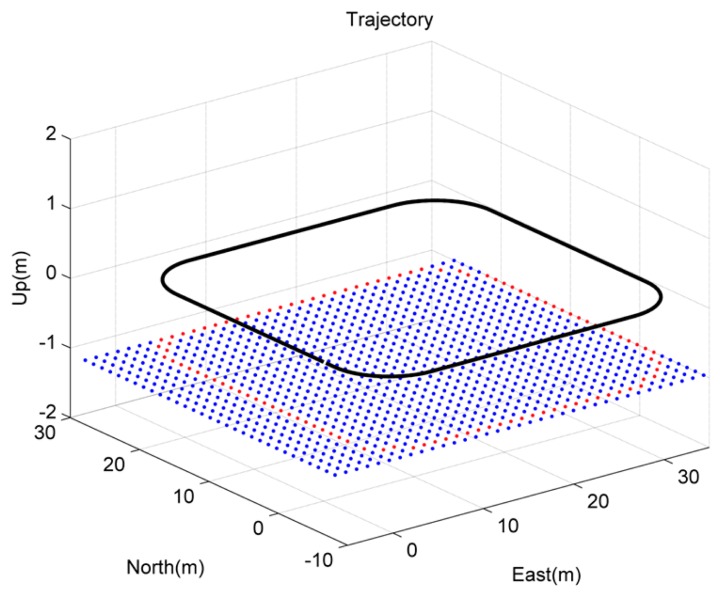
Simulated square trajectory of the camera/IMU-integrated platform and the layout of the feature points. columns show the errors of the estimated position and attitude, respectively. 3σ of the errors are marked by the dashed red line envelope.

It is uncommon that the camera can track the same feature points constantly in practice, because the field-of-view of the camera is limited. To test this, a third scenario was simulated with many known feature points deployed on the ground. As shown in Figure 11, the integrated camera/IMU platform was supposed to move in a square trajectory with the camera facing the ground where many known feature points are distributed. The platform was assumed to be 1 m above the ground. The performances of the sensors are the same as the sensors simulated in the first simulation. In the simulation, the feature points were projected onto the image plane and generated the image feature measurements. Then, the image measurements were superposed with randomly-generated white noise with a two-pixel standard deviation. The given coordinates of the feature points were added with randomly-generated white noise with a 1-cm standard deviation. It was assumed that only one feature point was observed in each image. This is shown in Figure 11, where the red feature points are the ones observed by the camera.

**Figure 12.**
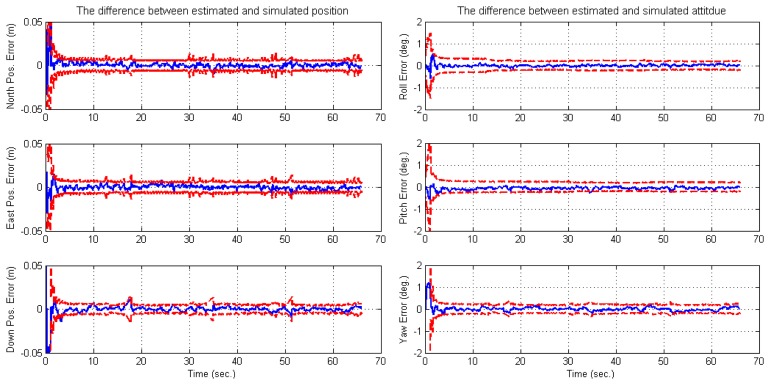
Estimation errors of the tightly-coupled image/INS solution for the third simulation. The first and second

For the situation that only one feature can be observed in each image, it is impossible for the loosely-coupled approach to manage the resulting data. However, the navigation system states are observable globally in the tight model, which can be referred to by the observability conclusion given in Section 3.2. This also yields the result that inertial errors are well bounded and calibrated (Figure 12). Furthermore, the statistic errors are better than 1 cm and 0.1 degree (RMS) for the position and attitude, respectively (Table 2).

### 5.2. Field Test

**Figure 13.**
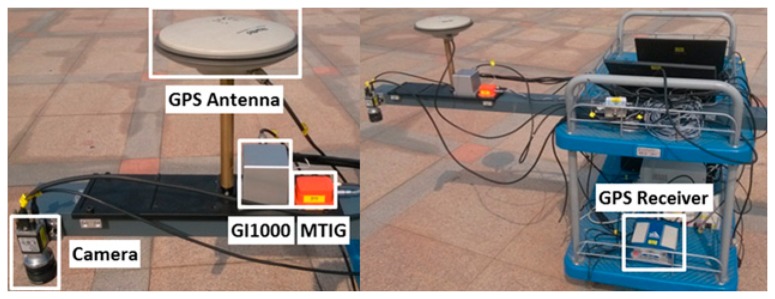
Sensors installation on a cart.

A field test of the GNSS/image/INS-integrated system was conducted at Wuhan University, China, on 3 April 2014. As shown in Figure 13, a cart equipped with various sensors was installed. To evaluate the integrated solution of image/INS, the result of the carrier-phase differential GPS (CDGPS)-aided INS was used as the reference solution. The sensor suite on the platform consists of the following sensors: (1) two six-degrees-of-freedom (6-DoF) IMUs, including a quasi-tactical GI-1000 IMU and a low-end MEMS MTi-G IMU; (2) a NovAtel DL-V3 GPS receiver that can output a double frequency pseudo-range, Doppler and carrier-phase measurements; (3) a Basler camera, which was downward-pointing at a height of 0.7 m above the ground. The camera exposure was hardware triggered by external pulses. The measurements from these three types of sensors were strictly time-synchronized and were precisely mounted on an aluminum alloy beam that was fixed to the cart. The alignment was guaranteed, and the lever-arms between the sensors were measured and calibrated in advance. The specifications of these sensors are given in Table 1. In the test, the IGS reference station, WUHN (Wuhan), located on campus, was also used as the GPS base station, forming a double-differenced CDGPS solution with the rover GPS measurements.

**Figure 14.**
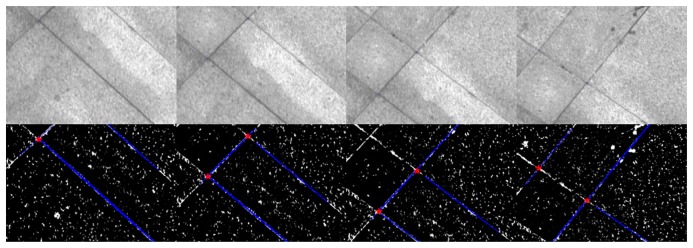
Feature points extracted from the images by intersecting the lines.

The field test was performed on a well-built square at the university. Feature points tracked by the camera came from the ground of the square directly. As shown in Figure 13, the ground is covered with regularly-shaped tiles that form many perpendicular lines on the ground. Because the cross points of the perpendicular lines are distributed evenly and easy to extract, the cross points were used as the known feature points that would be tracked in the images. During the data processing, the cross points were extracted by intersecting two perpendicular lines (Figure 14); the lines were detected using the method of “Hough transforms” [35].

The positions of the feature points are surveyed in advance using high-precision geodetic GPS receivers with a 4 h static observation. Because the feature points are in a plane and regularly distributed on the ground, only several feature points were surveyed. The positions of other feature points were obtained by interpolation. The baseline of the static GPS stations and IGS WUHN station are solved using GAMIT (GPS Analysis at MIT) [36]. Due to the 500-m length of the baselines between the base station and the rover stations, the accuracy of the baseline solution was at the millimeter level. The coordinates of the WUHN station are in the WGS84 coordinate system, which is an Earth-centered Earth-fixed coordinate system. After adjusting the baselines, the high-precision location of the feature points down to millimeter-level accuracy was obtained in the globally referenced WGS84 coordinate system (*i.e.*, the *e*-frame). However, the interpolating process contributed to a loss of precision to some degree, because the grids are not perfectly even in the real-world.

To match the extracted cross point from the image with the corresponding physical cross point, position information was used. First, the position of the camera center can be predicted using the current solution of the INS. The distance between the camera center and the physical cross point is also known (*i.e.*, the height of the camera above the ground). Thus, the position of the cross point in the *e*-frame can be predicted using Equation (29). Then, searching for the closest point in the position database will find the corresponding point. In addition, the update interval of the image measurements should be small enough to ensure that the errors of the INS solution are bounded. Otherwise, an incorrect feature point can be matched. The capture interval was set to 0.1 s, which is small enough to identify the correct cross point. As the smallest distance between two different cross points in the database is 0.2 m, during 0.1 s, INS errors cannot be larger than 0.2 m.

**Figure 15.**
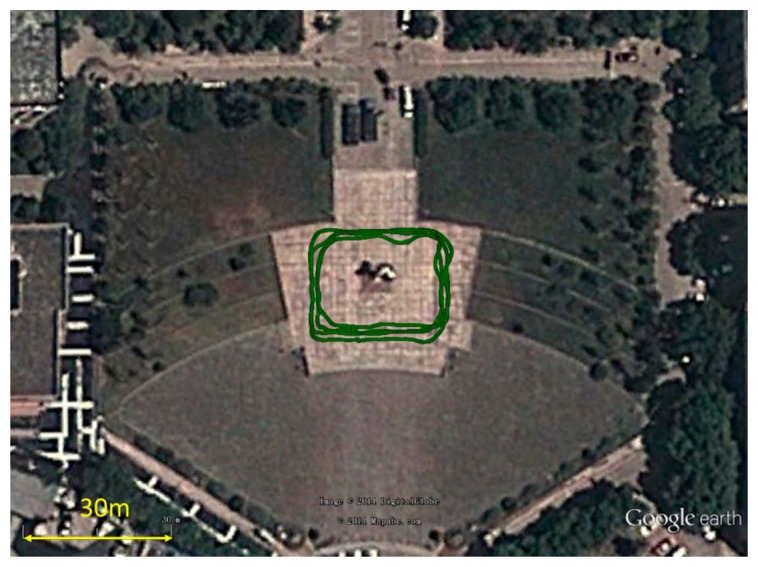
Trajectory of field test.

**Figure 16.**
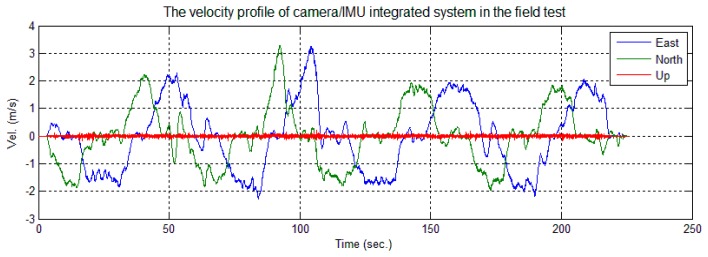
Velocity profile of the image/INS system.

**Figure 17.**
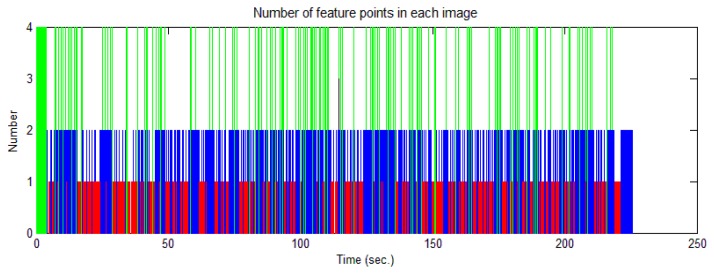
Number of feature points observed in the image sequence.

The cart was moved on the square along the trajectory shown in Figure 15. Figure 16 shows the velocity profile of the platform. The number of feature points observed changed in each image, as shown in Figure 17. Limited by the field-of-view of the camera, the largest number of feature points observed in an image was four; the detected number of feature points was mostly less than three. In this case, the tightly-coupled image/INS integration algorithm will be superior to the loosely-coupled method, which cannot consider the image measurements that contain only two or one feature point.

**Figure 18.**
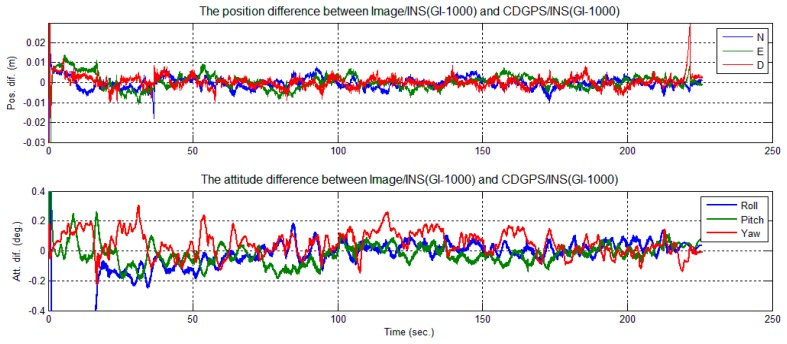
Position and attitude difference between image/INS (GI-1000) and CDGPS/INS.

**Figure 19.**
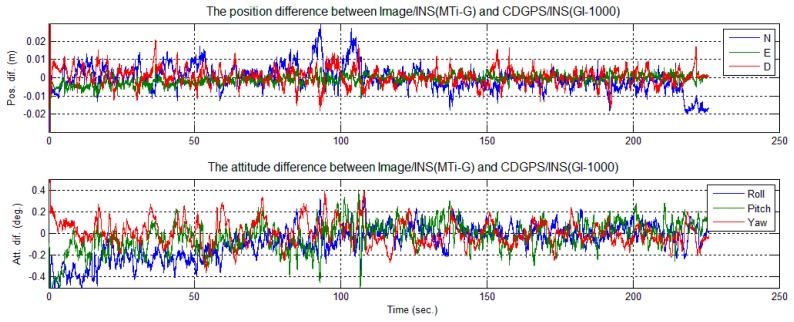
Position and attitude difference between image/INS (MTi-G) and CDGPS/INS.

To evaluate the performance of the tightly-coupled image-aided INS-integrated solution, a backward smoothing solution of CDGPS-aided INS was chosen as the reference solution. The position and attitude differences between the image/INS and the reference are shown in Figure 18 for GI-1000 and in Figure 19 for MTi-G.

A few error spikes appear at the end of the solution difference curves (Figures 18 and 19). As shown, the down position difference increases to a maximum value of 0.03 m and falls to a normal value quickly. In this short period, the cart was almost static and only one feature point was observed, as shown in Figures 16 and 17. According to Theorem 1, the scale factors *λ*_1_ were unobservable when only one feature point was observed, and its location changed minutely in the images. Therefore, the solution could be considered to be drift with INS sensor bias. The cart then moved slightly; two feature points were observed, and their locations changed in the images. According to Theorem 2, the integrated system became observable again, and the difference of the solution reduced immediately. Therefore, this phenomenon also confirms the observability analysis conclusions given in Section 3.

**Figure 20.**
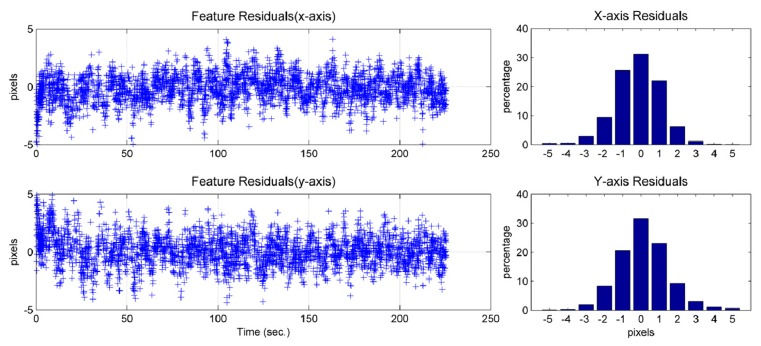
Feature residual of the image/INS (GI-1000) solution and its percentage histograms.

**Figure 21.**
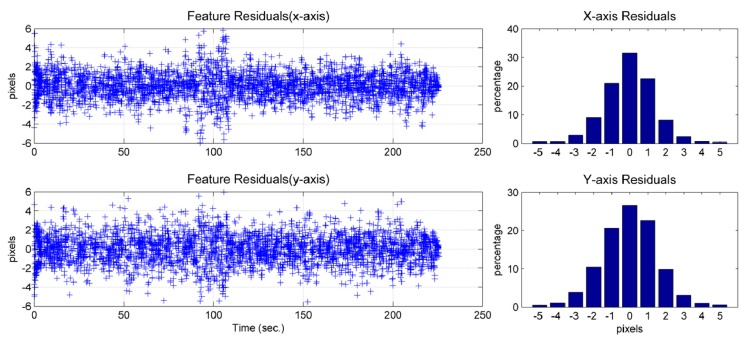
Feature residual of the image/INS (MTi-G) solution and its percentage histograms.

The feature residual is the difference between the measurement of a pixel feature location and the prediction of the pixel feature location using the image/INS state estimates (*cf.* (4)). This obeys a normal distribution (Figures 20 and 21) and provides insight into the precision that the solution can achieve. Due to the characteristics of the camera used, one pixel in the image would represent approximately 4 mm, approximately, in the real world on the ground. Because most of the feature residuals are smaller than five pixels (Figures 20 and 21), it can be inferred that the position precision of the image-aided INS should be better than 2 cm.

The differences between the solution and the reference attitude error are less than 0.5 degrees (Figures 18 and 19). It can be observed that the attitude solution using MTi-G is only slightly nosier than that using GI-1000 (Tables 3 and 4), although the sensor performance of MTi-G is much worse than that of GI-1000 (Table 1). This is because the integrated attitude accuracy strongly depends on the accuracy that the image observations can provide.

Compared to the conventional GNSS/INS integration system, the observable conditions of the attitude are different for image-aided INS and CDGPS-aided INS systems. For both of the systems, the roll and pitch error could be directly determined by measuring the gravity vector. However, for GNSS-aided INS, the yaw angle will become observable when the vehicle is under accelerating [19]. For the tightly-coupled image-aided INS, one line-of-sight observation can only provide observable information along the directions perpendicular to the feature line-of-sight [10]. Additionally, the line-of-sight measurements in these tests spanned the 3D space, because the locations of the features always changed on the image plane, ensuring that the yaw angle was observable. From the observability point of view, the image aiding for INS is complementary to the GNSS aiding, to some extent.

## 6. Conclusions

A tightly-coupled image-aided inertial navigation system has been developed and analyzed for observability from a global perspective.

The observability analysis reveals that tracking two known feature points that are not in a vertical line can ensure that the states of the integrated system remain observable as long as the locations of both feature points on the image plane change with time. In the case that different known feature points are tracked, observing only one feature point at a time can ensure that the navigation states of the system are observable, as long as the locations of the feature points on the image plane change. Additionally, if estimation of the accelerometer bias is required, then the system should rotate about at least two axes of the *b*-frame simultaneously.

Simulation and field test evaluations have shown that the position precision of the proposed tightly-coupled image-aided INS is better than two centimeters in a close-range distance, even with only one feature point available in the images. The attitude precision of the system is shown to be better than 0.5 degrees. Thus, this image-aided INS can be applied as a high-precision positioning technology in a GNSS-denied environment.

Future work includes: tightening the given sufficient conditions of global observability of the camera/IMU-integrated system in this paper to become sufficient and necessary conditions, by considering that the magnitude of the local gravity is known; evaluating the proposed system in a more professional test field; and improving the precision of the proposed system by using a high-grade IMU. The authors would also like to explore the potential of using the proposed system as a reference to evaluate other high-precision navigation systems in dynamic situations.

## Figures and Tables

**Table 1.** Specifications of sensors.

**Sensor**	**Parameter**	**Value**
**IMU (GI-1000)**	Angular Random Walk (ARW)	0.2 deg/√h
	Gyro Bias Instability	7 deg/h
	Velocity Random Walk (VRW)	0.18 m/s/√h
	Accelerometer Bias Instability	400 mGal

**IMU (MTi-G)**	Angular Random Walk (ARW)	3 deg/√h
	Gyro Bias Instability	200 deg/h
	Velocity Random Walk (VRW)	0.12 m/s/√h
	Accelerometer Bias Instability	2000 mGal

**Camera (Basler)**	Resolution horizontal/vertical	1,628 pixels × 1,236 pixels
	Pixel Size horizontal/vertical	4.4 μm × 4.4 μm
	Frame Rate	10 fps
	Focal length	8.5 mm

**Table 2.** Statistical summary of tightly-coupled image/INS solution error.

**Statistical Value**	**Position Error (m)**			**Attitude Error (degree)**		
	
	**North**	**East**	**Down**	**Roll**	**Pitch**	**Yaw**
Mean	0.0001	−0.0002	0.0001	−0.0043	−0.0612	−0.0105
Rms	0.0020	0.0021	0.0023	0.0410	0.0773	0.0693
Max	0.0102	0.0096	0.0114	0.1391	0.2644	0.2109

**Table 3.** Statistical summary of solution differences between image/INS (GI-1000) and carrier-phase differential GPS (CDGPS)/INS.

**Statistical Value**	**Position Difference (m)**			**Attitude Difference (degree)**		
	
	**North**	**East**	**Down**	**Roll**	**Pitch**	**Yaw**
Mean	−0.0004	0.0001	0.0002	−0.0043	−0.0612	−0.0105
Rms	0.0024	0.0028	0.0027	0.0681	0.0630	0.0861
Max	0.0180	0.0108	0.0294	0.2509	0.1976	0.3076

**Table 4.** Statistical summary of solution difference between image/INS (MTi-G) and CDGPS/INS.

**Statistical Value**	**Position Difference (m)**			**Attitude Difference (degree)**		
	
	**North**	**East**	**Down**	**Roll**	**Pitch**	**Yaw**
Mean	−0.0004	−0.0006	0.0004	−0.0515	−0.0106	−0.0006
Rms	0.0065	0.0023	0.0043	0.1253	0.1112	0.1052
Max	0.0298	0.0114	0.0208	0.4220	0.4963	0.3937
